# The Vascular-Dependent and -Independent Actions of Calcitonin Gene-Related Peptide in Cardiovascular Disease

**DOI:** 10.3389/fphys.2022.833645

**Published:** 2022-02-25

**Authors:** Fulye Argunhan, Susan D. Brain

**Affiliations:** Section of Vascular Biology & Inflammation, School of Cardiovascular Medicine & Research, BHF Centre of Excellence, King’s College London, London, United Kingdom

**Keywords:** CGRP, cardiovascular, heart, mouse, nitric oxide, hypertension, heart failure

## Abstract

The treatment of hypertension and heart failure remains a major challenge to healthcare providers. Despite therapeutic advances, heart failure affects more than 26 million people worldwide and is increasing in prevalence due to an ageing population. Similarly, despite an improvement in blood pressure management, largely due to pharmacological interventions, hypertension remains a silent killer. This is in part due to its ability to contribute to heart failure. Development of novel therapies will likely be at the forefront of future cardiovascular studies to address these unmet needs. Calcitonin gene-related peptide (CGRP) is a 37 amino acid potent vasodilator with positive-ionotropic and -chronotropic effects. It has been reported to have beneficial effects in hypertensive and heart failure patients. Interestingly, changes in plasma CGRP concentration in patients after myocardial infarction, heart failure, and in some forms of hypertension, also support a role for CGRP on hemodynamic functions. Rodent studies have played an important role thus far in delineating mechanisms involved in CGRP-induced cardioprotection. However, due to the short plasma half-life of CGRP, these well documented beneficial effects have often proven to be acute and transient. Recent development of longer lasting CGRP agonists may therefore offer a practical solution to investigating CGRP further in cardiovascular disease *in vivo*. Furthermore, pre-clinical murine studies have hinted at the prospect of cardioprotective mechanisms of CGRP which is independent of its hypotensive effect. Here, we discuss past and present evidence of vascular-dependent and -independent processes by which CGRP could protect the vasculature and myocardium against cardiovascular dysfunction.

## Introduction

The discovery of Calcitonin gene-related peptide (CGRP) mRNA in the rat hypothalamus by Amara et al. (1982) sparked a series of studies exploring the effect of CGRP in the central and peripheral systems, where it is widely distributed. The authors discovered that human CALCA gene, which codes for the thyroid gland hormone calcitonin, can also produce CGRP *via* alternative splicing in neural tissues. CGRP is a member of the calcitonin family of peptides, that include adrenomedullin, adrenomedullin 2/intermedin and amylin (Russell et al., 2014). Soon after, it was found that CGRP has two structurally similar isoforms – the α and β, which are encoded by two distinct genes (Amara et al., 1985). Both isoforms are primarily located in sensory C- and Aδ-fibers (Gibson et al., 1984), and it is generally accepted that despite 94% sequence identity between the two isoforms, β-CGRP synthesis and expression is concentrated around the enteric nervous system, immune cells and pituitary gland (Steenbergh et al., 1985; Brain and Grant, 2004) whereas α-CGRP is primarily involved in the central and peripheral nervous systems, and consequently, is the more extensively studied isoform in cardiovascular studies. This review aims to discuss past and present literature on CGRP in hypertension and heart failure to stimulate thought on the future of CGRP and cardiovascular research, with a particular focus on α-CGRP.

The current consensus is that, upon neuronal depolarization, α- or β-CGRP is released from sensory neurons *via* calcium-dependent exocytosis to bind to its receptor situated on the plasma membrane of several cell types including, but not limited to, smooth muscle cells (Argunhan et al., 2021), endothelial cells (Gray and Marshall, 1992a) and cardiomyocytes (Clark et al., 2021). The receptor complex is composed of a seven domain G-protein coupled receptor (GPCR) known as calcitonin receptor-like receptor (CRLR) and a single transmembrane protein recognized as receptor activity modifying protein-1 (RAMP1). RAMP1 is required for trafficking of the receptor to the cell surface to form a heterodimer with CRLR and mediate high-affinity binding to CGRP. Upon activation of the CRLR/RAMP1 receptor, G-protein induced signaling cascade is initiated, with Gα_s_-induced cyclic adenosine monophosphate (cAMP) being the major secondary messenger involved (McLatchie et al., 1998; Pioszak and Hay, 2020). The CGRP family of receptors also comprise two other RAMP proteins; RAMP2 and RAMP3. There is evidence that CGRP may be able to signal *via* receptors including these components. The receptors involved comprise the calcitonin receptor (CTR) interacting with RAMP1, commonly called the amylin receptor and CRLR interacting with RAMP2, the adrenomedullin receptor (Hay et al., 2018). The selectivity of CGRP for receptors other than CRLR/RAMP1 is under investigation.

**FIGURE 1:**
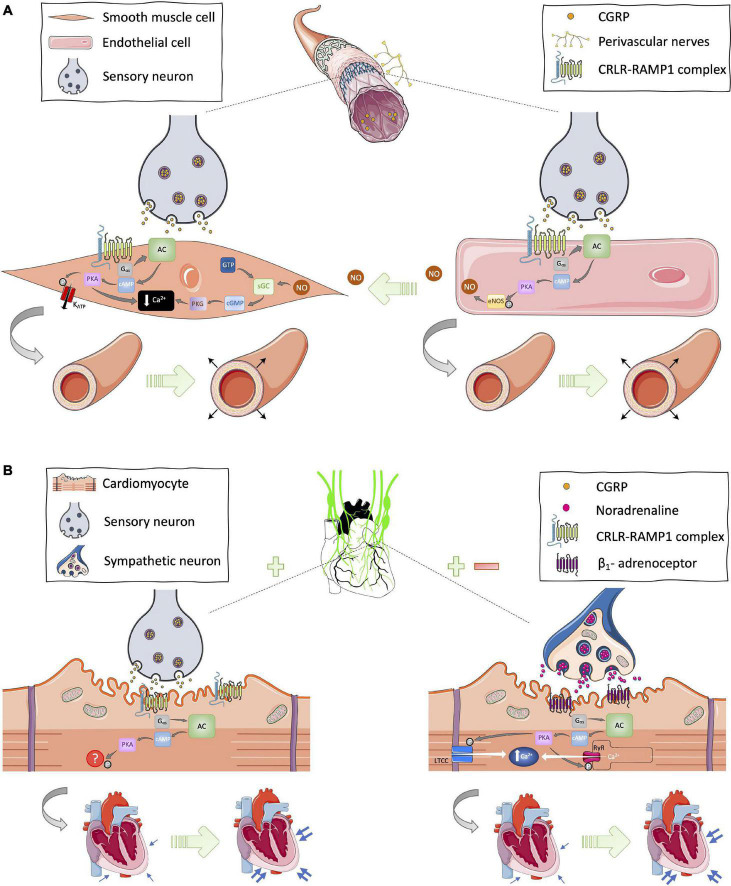
Proposed cardioprotective mechanisms of CGRP in the **(A)** vasculature and **(B)** myocardium. **(A)** In the vasculature, CGRP circulating in the blood or released from sensory neurons can bind on to its canonical receptor, the CRLR-RAMP1 complex, expressed in the plasma membrane of vascular smooth muscle and endothelial cells to initiate Gα_s_-protein signal transduction and subsequently cause relaxation of smooth muscle cells *via* nitric oxide (NO)-dependent and -independent mechanisms thus leading to vasodilation. **(B)** In cardiac tissues, there is evidence for CGRP-stimulated modulation of sympathetic outflow and expression of the CRLR-RAMP1 complex on cardiomyocytes. Hence, CGRP has the potential to induce Gα_s_-protein signaling from sensory and sympathetic nerves leading to increased cardiac contractility, thus positive inotropy and chronotropy. AC, adenyl cyclase; cAMP, cyclic adenosine monophosphate; cGMP, cyclic guanosine monophosphate; eNOS, endothelial nitric oxide synthase; GTP, guanosine triphosphate; K_ATP_, ATP-sensitive potassium channels; LTCC, L-type calcium channel; NO, nitric oxide; P, phosphate; PKA, protein kinase A; RyR, ryanodine receptor; sGC, soluble guanylate cyclase. [Images were obtained from smart.servier.com under a Creative Commons Attribution 3.0 Unported License].

Due to the CRLR/RAMP1 receptor complex being expressed in the plasma membrane of smooth muscle cells and endothelial cells, CGRP-induced vasodilation can manifest *via* two distinct but related mechanisms. The CGRP ligand can bind directly to the receptor complex in vascular smooth muscle cells to induce PKA-mediated smooth muscle relaxation, or it can interact with its receptor complex in endothelial cells to induce endothelial-dependent relaxation *via* nitric oxide signaling (Crossman et al., 1990; Gray and Marshall, 1992b). In vascular smooth muscle cells, CGRP-induced PKA phosphorylation leads to: reduced intracellular Ca^2+^ concentration; reduced binding affinity of myosin light chain kinase (MLCK) to Ca^2+^-calmodulin complex; and activation of ion channels such as ATP-sensitive potassium channels (K_ATP_). All of these contribute to smooth muscle cell relaxation, and thus vasodilation (Brain and Grant, 2004; Figure 1A). In endothelial cells, PKA phosphorylation of eNOS results in NO generation, which is known to diffuse into neighboring vascular smooth muscles and mediate smooth muscle cell relaxation *via* guanyl cyclase and protein kinase G (PKG) signaling induction pathways (Russell et al., 2014; Figure 1A). Additionally, there is some evidence for PKC-mediated responses post-CGRP receptor activation (Walker et al., 2010) but the cAMP response is the most established and understood signaling pathway to date. However, it is important to acknowledge the body of *in vitro* evidence suggesting that the CGRP receptor can couple with other G proteins (Weston et al., 2016). It is also noteworthy that, in addition to its canonical receptor, CGRP has been shown to interact with other receptors from the CGRP family of peptides, albeit with lower affinity (Hay et al., 2018). Recently, the structure and dynamics of the canonical CGRP receptor has been investigated using single-particle cryo-EM (Liang et al., 2018; Josephs et al., 2021), and agonist bias studies have revealed physiological consequences for the CRLR-RAMP1 complex in different cell types (Clark et al., 2021). Thus, this is an area of much interest that is continually evolving, with RAMP proteins being the subject of investigation.

## Calcitonin Gene-Related Peptide and Vascular Tone – Small but Mighty

Fisher et al. (1983) were the first to demonstrate the hypotensive effects of systemically administered CGRP in rats, whereas Brain et al. (1985, 1986) showed that intradermal injection of CGRP at femtomole doses induces arteriole dilation, thus increasing blood flow locally in animal and human skin. These studies and others indicated that CGRP is a potent vasodilator, leading researchers to further explore its beneficial potential within the cardiovascular system. As a microvascular vasodilator, the potency of CGRP is approximately 10-fold higher than prostaglandins, up to 100 times greater than other well-established vasodilators such as acetylcholine, thus making CGRP the most potent peripheral vasodilator discovered to date (Russell et al., 2014).

Gennari and Fischer (1985) were first to determine the cardiovascular actions of CGRP on hemodynamic parameters in healthy humans. In addition to CGRP’s intrinsic ability to cause vasodilation and consequently hypotension, the authors revealed that CGRP possesses positive chronotropic and inotropic activities. CGRP was reported to increase the force of contractility by stimulating the sympathetic nervous system, which was further supported by Gennari et al. (1990) when patients with congestive heart failure (CHF) demonstrated improved cardiac contractility after receiving β-CGRP infusion for 24 h (12.5 ug/h). *Ex vivo* experiments reinforce these findings, although the mechanism behind the positive inotropic effect is still to be fully elucidated. Franco-Cereceda et al. (1987) were the first to report the effect of CGRP treatment in isolated human auricles, and interestingly, the authors found both α- and β- isoforms of CGRP to be equally potent in affecting positive inotropy.

Similarly, Struthers et al. (1986) infused hCGRP into healthy patients (545 pmol/min), which, as expected, caused a significant decrease in diastolic pressure accompanied with an increase in heart rate. The authors suggested a potential role for CGRP in modulating vascular tone in humans, and this was supported by findings from Girgis et al. (1985) demonstrating that CGRP concentration is fivefold higher than calcitonin. Given that it is widely expressed in the human body, these results raised the possibility that CGRP is a critical physiological regulator of vascular tone and hemodynamics.

Collectively, these studies laid the foundation for elucidating the biological actions of CGRP, which recently reached fruition in the field of migraine research where CGRP blockers are now used therapeutically (Edvinsson et al., 2018; Kee et al., 2018). Generally, the use of migraine blockers have not been associated with significant cardiovascular effects, indicating that CGRP does not play a major role in cardiovascular regulation in individuals with normal blood pressure (Bigal et al., 2015). Separately, there has been increasing research into elucidating the role of CGRP and of exogenous CGRP, in the cardiovascular system. However, the potential biological importance and underlying mechanisms, particularly with respect to heart failure, will be discussed in later sections.

## Calcitonin Gene-Related Peptide in Hypertension – Vascular-Dependent Effects

Shekhar et al. (1991) was one of the first to demonstrate the cardiovascular effects of prolonged CGRP infusion (8 ng/kg/min for 8 h) in CHF patients. In agreement with others, they reported significant decreases in cardiac and arterial pressures, as well as in pulmonary and systemic vascular resistance, and increases in cardiac output and stroke volume. Accordingly, the reported hemodynamic changes were absent 30 min after discontinuation of CGRP infusion, consistent with the short half-life of CGRP (approximately 7–30 min) (Kraenzlin et al., 1985).

Concomitantly, preclinical data from rodents have facilitated human studies. Research in the 1990s focusing on the role of CGRP in blood pressure control was dominated by preclinical hypertension studies carried out in rats that were spontaneously hypertensive (SHR), treated with hypertensive agents, or hypertensive due to surgical intervention. These studies and others reported *in vivo* evidence that CGRP can protect against hypertension and the vasoconstrictor effects of hypertensive agents including angiotensin-II (AngII) (Itabashi et al., 1988; Fujioka et al., 1991), noradrenaline (Fujioka et al., 1991), deoxycorticosterone-salt (Supowit et al., 1997; Supowit et al., 2005), and the nitric oxide synthase (NOS) inhibitor L-NAME (Gardiner et al., 1991; Gangula et al., 1997), making progress toward understanding the mechanism of action of CGRP (Kumar et al., 2019a). Kawasaki et al. (1990) found that reduced CGRP-containing nerves in SHRs contribute to the development and maintenance of hypertension, further supporting a protective role for CGRP in hypertension. Furthermore, *ex vivo* experiments, particularly in isolated arterial and mesenteric resistance vessels, have complemented the *in vivo* data to-date (Nelson et al., 1990; Kawasaki et al., 1998; Kawasaki et al., 1999).

It has been difficult to study α-CGRP *in vivo* due to its peptide nature, therefore the use of genetically engineered mice has been fitting. Genetic deletion of CGRP has been reported to cause elevated baseline blood pressure in some (Gangula et al., 2000; Oh-hashi et al., 2001; Li et al., 2004; Mai et al., 2014) but not all studies (Smillie et al., 2014; Argunhan et al., 2021). This is likely due to differences in methodology, such as differences in the precise genetic deletion site when generating knockout (KO) mouse lines, particular strains preserving unique hemodynamic phenotypes, and the utilization of various different blood pressure measurement techniques. Most importantly, most preclinical hypertension studies conclude that genetic deletion of CGRP is detrimental, if not in a naïve state, then evidently in a stressed or hypertensive setting. This indicates that endogenous CGRP may only be functionally active in cardiovascular dysfunction. CGRP blockers (antibodies and now CGRP receptor antagonists) have been studied in humans with migraine over several years now (Al-Hassany and Van Den Brink, 2020). The majority of findings from recent clinical trials report little or no change in blood pressure from migraine patients who received anti-CGRP therapy. Additionally, very few cardiovascular side-effects have been observed in migraine patients taking CGRP blockers (Tepper, 2019). Thus, it is not yet known whether CGRP has a functionally important cardiovascular role in humans, apart from the neurogenic vasodilator response observed typically in patients with migraine (Tepper, 2019). However, due to CGRP being widely expressed and the increasing evidence for a cardioprotective role, there are concerns regarding long-term blockade of CGRP in migraine patients who may also suffer from cardiovascular complications (Rubio-Beltran and Maassen van den Brink, 2019). The observation of cardiovascular adverse effects with CGRP blockers in migraine has not been commonly observed. However, a recent study conducted a retrospective analysis of cases reporting a CGRP receptor antagonist (erenumab) associated with elevated blood pressure (BP) (Saely et al., 2021). The authors identified 61 cases of elevated BP between May 2018 and April 2020, of which the median systolic BP increase was 39 mm Hg. Interestingly, 44% of reported cases required anti-hypertensive medication and the elevated BP occurred most frequently within a week of commencing erenumab treatment. Most importantly, the prescribing information for erenumab/Aimovig now includes hypertension. This further supports the need to continue monitoring cardiovascular parameters in those receiving anti-CGRP therapy for migraine, in addition to continuing research within the CGRP and cardiovascular field (Saely et al., 2021).

Much like CGRP KO mice, RAMP1 KO mice develop high blood pressure (Tsujikawa et al., 2007) and knock-in or overexpression of human RAMP1 in all (Sabharwal et al., 2010) or solely neural tissues (Sabharwal et al., 2019) potentiates CGRP-dependent blood pressure reduction in AngII-induced hypertension. Additionally, in the periphery, in tissues such as skin, it is clear that CGRP is a potent vasodilator and is well placed to play a regulatory role, for example in the recovery of blood flow in the cold-induced vascular response (Aubdool et al., 2014), a response that diminishes as aging occurs (Thapa et al., 2021).

Our research group has reported that, despite a lack of vascular tone modulation at baseline, α-CGRP-specific KO mice display elevated blood pressure after AngII or L-NAME treatment (Smillie et al., 2014; Argunhan et al., 2021). α-CGRP KO mice present with hypertrophic vascular remodeling in their aortic tissues and increased mRNA levels of inflammatory and oxidative stress markers after 14 or 28 days of AngII treatment (Smillie et al., 2014). These results indicate a protective role for CGRP in AngII-induced pathophysiology, where the benefits are not limited to one system, but instead multiple processes in order to attenuate the pathophysiological changes induced by AngII.

Intriguingly, Smillie et al. (2014) also showed that AngII-treated α-CGRP KO mice display a significant reduction in protein and mRNA expression of endothelial nitric oxide synthase (eNOS). These findings probed us to investigate whether α-CGRP can protect against hypertension independently of eNOS *in vivo.* The decreased production of NO in pathological states disrupts the endothelial equilibrium thus leading to endothelial dysfunction. CGRP receptors are expressed in the plasma membrane of both endothelial and vascular smooth muscle cells, hence have the capacity to induce vasodilation *via* NO-dependent and -independent pathways. However, there is very limited evidence of this *in vivo.* We demonstrated that α-CGRP KO mice develop exacerbated hypertension and present with dysfunctional blood flow recovery in mesenteric vessels *in vivo* after chronic L-NAME administration (Argunhan et al., 2021) indicating that α-CGRP can induce vasodilation and hence attenuate hypertension independently of NOS. These findings suggest that α-CGRP may be able to offer protection to compensate for pathophysiological processes such as endothelial dysfunction, which contributes to hypertension and cardiovascular disease. We also found that 2 weeks of α-CGRP infusion (165 ug/kg/day) *via* osmotic minipumps was able to reverse L-NAME-induced hypertension, left ventricular heart weight gain, and associated increases in mRNA expression of hypertrophic markers in α-CGRP KO mice, providing evidence for the antihypertrophic effects of CGRP. Conversely, this means that the nitric oxide vasodilator pathway theoretically could compensate, in terms of vasodilation, when CGRP is inhibited.

Antihypertrophic effects of CGRP have also been investigated recently by Skaria et al. (2019). The authors examined whether endogenous, physical activity-induced α-CGRP has blood pressure-independent cardioprotective effects in mice which had 1 kidney 1 clip (1k1c) surgery and hence developed chronic hypertension. The authors claimed that exercise has cardioprotective effects in chronic hypertension, which is mediated at least partially through endogenous α-CGRP signaling. They demonstrated αCGRP concentration in plasma is significantly elevated after 7 min of running in hypertensive mice and showed that chronic exogenous CGRP infusion *via* osmotic minipumps can alleviate hypertension-induced hypertrophy and cardiac dysfunction by suppressing pathological cardiac growth and interstitial fibrosis. Importantly, CGRP was infused at a sub-pressor dose (4 nM/h), suggesting that CGRP administration can help to preserve cardiac function in chronic hypertension independent of its blood pressure lowering effect.

## Long-Lasting Agonist of Calcitonin Gene-Related Peptide

While the protective effects of CGRP discussed thus far are detailed and mostly consistent between research groups, they do not constitute a complete record of documented studies demonstrating a cardioprotective role of CGRP. It is apparent that the beneficial effects of CGRP have been limited due to its short peptide half-life. Our group was fortunate to investigate the therapeutic potential of a longer lasting CGRP agonist in AngII-induced hypertension and mice that underwent abdominal aortic constriction surgery, which eventually caused heart failure *via* increased pressure-overload on the heart (Aubdool et al., 2017). The acylated α-CGRP analog had been characterized by Nilsson et al. (2016) and has a half-life of >7 h in rodents. Our group demonstrated that daily administration of the CGRP analog [50 nmol/kg/day, *subcutaneous* injection *(s.c.)]* for 2 weeks in AngII-treated mice led to significant attenuation of AngII-induced hypertension and protected against vascular, renal and cardiac dysfunction. α-CGRP analog-treated mice presented with attenuated hypertrophic and fibrotic markers as well as reduced inflammation and oxidative stress. Furthermore, the α-CGRP analog was effective in preserving ejection fraction, a measure of cardiac function, and protecting against fibrosis and apoptosis in cardiac tissues of mice that had undergone abdominal aortic constriction surgery and consequently developed heart failure. Moreover, α-CGRP-treated mice presented with better vascularization in their hearts and expressed reduced mRNA and protein expression of biomarkers for hypertrophy, apoptosis, oxidative stress and inflammation. These findings agree with other *in vivo* heart failure studies (Li et al., 2013; Kumar et al., 2019b; Kumar et al., 2020). *In vitro* experiments have demonstrated similar findings in different cell types. CGRP administration has been shown to: stimulate proliferation of endothelial cells (Haegerstrand et al., 1990), supporting CGRP’s proangiogenic effects *in vivo*; reduce vascular smooth muscle cell proliferation (Li et al., 1997) and thus vascular hypertrophy; and show antioxidant and antiapoptotic effects in dorsal root ganglion (DRG) neurons (Liu et al., 2019).

However, data from the Aubdool et al. (2017) study suggests these protective effects of CGRP can be long lasting using a novel α-CGRP agonist with an extended half-life. The CGRP agonist can delay the onset and development of hypertension through cardioprotective mechanisms in addition to ameliorating pressure overload-induced heart failure. Interestingly, mice with heart failure that had received the α-CGRP analog or its vehicle presented with comparable blood pressures, indicating that the cardioprotective mechanisms involved are likely to be blood pressure-independent. Furthermore, the same analog has recently been administered to rats that have undergone permanent occlusion of their left coronary artery to investigate coronary perfusion in myocardial infarction. Three injections of the CGRP analog, SAX, at 20 min, 24 and 48 h after coronary ligation, was sufficient to improve myocardial perfusion recovery in rats, indicative of myocardium protection after ischemic damage (Bentsen et al., 2021). An earlier study by Kallner et al. (1998) reported CGRP-mediated improvement in post-ischemic coronary flow early after MI, but whether treatment with the long lasting agonist will increase cardioprotective effects in the long term is still to be investigated.

## Calcitonin Gene-Related Peptide in the Myocardium – Vascular-Independent Effects

The heart is densely innervated by nerve fibers comprised of: the sympathetic trunk, which starts from the base of the skull; parasympathetic nerves, including the right and left vagus nerves; cervical cardiac nerves, which run parallel to the vagus nerves; and the cardiac plexus at the base of the heart. α-CGRP producing sensory nerves have been reported within the perivascular layer of coronary arteries, in the myocardium of ventricles, and within the cardiac conduction system (Gulbenkian et al., 1993). In addition to this, CGRP immunoreactivity has been associated with; myocytes from atria, coronary vessels, local parasympathetic ganglia and with epi-and endocardia (Franco-Cereceda et al., 1987). Therefore, it is likely that CGRP signaling manifests within cardiac cells, in addition to cardiac vessels, and that the sensory, parasympathetic and sympathetic nerves are involved in facilitating CGRP-induced signaling within, and surrounding, the myocardium.

Several *in vivo* studies of hypertension and heart failure have reported an upregulation of RAMP1 and/or CGRP expression in pathological conditions (Supowit et al., 1995; Li and Wang, 2005; Aubdool et al., 2017; Argunhan et al., 2021). In addition to this, Franco-Cereceda and Liska (2000) have previously reported the presence of a subpopulation of capsaicin-sensitive cardiac C-fiber afferents that store CGRP, substance P and neurokinin A. The C-fibers are likely to express transient receptor potential vanilloid 1 (TRPV1) channel, which upon stimulation by capsaicin lead to CGRP release (Franco-Cereceda, 1991). This finding of local efferent release of CGRP in the heart is consistent with the presence of capsaicin-sensitive receptors on the epicardial surface of rat hearts (Zahner et al., 2003). More recently, Moreira et al. (2020) reported elevated CGRP levels in human atrial tissue lysates and atrial cardiomyocytes obtained from patients with atrial fibrillation, in agreement with Franco-Cereceda et al. (1987) who found between threefold and fourfold higher levels of CGRP-like immunoreactivity in atria compared to ventricles. These studies describe and support a structural basis for CGRP signaling within cardiac tissues.

Furthermore, coinciding with its interactions in vascular smooth muscle and endothelial cells, CGRP has similarly been demonstrated to bind to its canonical receptor complex and activate Gα_s_-signaling in cardiomyocytes (Huang et al., 1999; Sueur et al., 2005; Schavinski et al., 2021). It is well established that an increase in cAMP concentration followed by PKA activation leads to phosphorylation of key Ca^2+^-handling proteins including phospholamban, ryanodine receptor, voltage-gated L-type Ca^2+^ channels, troponin I, and myosin binding protein C; all which play an essential role in cardiac excitability and contraction (Zaccolo, 2009). Earlier studies have reported that activation of the CRLR/RAMP1 complex leads to stimulation of a contractile response in adult rat ventricular cardiomyocytes (Bell and McDermott, 1994; Bell et al., 1995). However, unlike the vascular-dependent effects of CGRP, the precise mechanism of action of CGRP in cardiomyocytes remains to be fully elucidated.

Interestingly, a recent study investigating GPCR agonist bias in CGRP and CGRP-like family peptides demonstrated that, in human ventricular cardiomyocytes, CGRP is more potent than adrenomedullin and adrenomedullin 2/intermedin in generating cAMP (Clark et al., 2021). Acute cAMP elevation is known to compensate for impaired cardiac function by modulating the positive-inotropic, -chronotropic and -lusitropic responses in the heart. Thus, in addition to its vascular dependent actions, it is tempting to speculate that CGRP-induced positive-inotropy and -chronotropy observed in earlier human studies could be a consequence of vascular-independent actions *via* direct interaction with cardiomyocytes. Chronic cAMP activation, however, has been associated with adverse cardiac remodeling (Triposkiadis et al., 2009). Importantly, cAMP is a pleiotropic secondary messenger and thus able to produce several biological outcomes in response to different stimuli. Moreover, locally accumulated cAMP has been shown to ameliorate cardiac hypertrophy *via* cAMP-degrading enzyme phosphodiesterase-2 (PDE2; Zoccarato et al., 2015). The regulation and function of local cAMP-PKA signaling remains to be fully understood, and compartmentalized cAMP-PKA has been suggested to play a key role in cardiac physiology and pathophysiology (Surdo et al., 2017).

Coupling of the CRLR-RAMP1 complex to Gα_s_ can lead to other responses such as phosphorylation of ERK1/2 or cell proliferation; a response which has been suggested to be cell type dependent (Clark et al., 2021). Interestingly, the CGRP family of receptors, including CRLR/RAMP1, can couple to Gα_i_ and Gα_q_ subunits too, although there is little evidence of this in cardiomyocytes (Nishikimi et al., 1998; Walker et al., 2010). These findings further support the need to investigate CGRP-signaling in cardiomyocytes and fibroblasts *in vitro*, which may help to clarify the mechanism of action behind the cardioprotective effects of CGRP reported in whole body physiology studies (Li et al., 2013; Aubdool et al., 2017; Kumar et al., 2019b; Skaria et al., 2019; Kumar et al., 2020; Argunhan et al., 2021), especially in studies that have shown blood pressure independent effects of CGRP (Aubdool et al., 2017; Skaria et al., 2019).

In addition to its direct effect on cardiomyocytes, CGRP has also been shown to modulate sympathetic nervous activity (Figure 1B). Activation of β-adrenoceptors *via* increased sympathetic nervous activity leads to Gα_s_-signaling-induced cardiac contractility. Earlier studies suggest that CGRP-induced positive inotropic effects may be, at least partially, due to increased sympathetic activity (Fisher et al., 1983; Gennari and Fischer, 1985; Struthers et al., 1986; Katori et al., 2005). On the other hand, Kawasaki (2002) reported that exogenous CGRP treatment can impair noradrenergic-induced constriction in rat mesenteric vessels. Thus, it is thought the activation or increased sympathetic activity reported in some studies is part of a compensatory reflex system to combat CGRP-induced hypotension. Considering that exogenous administration of CGRP primarily lowers blood pressure, its ability to stimulate the sympathetic nervous system is likely to be minimal and secondary to its primary action of inducing vasodilation. Evidence suggests that stimulation of the sympathetic nervous system may be one of the mechanisms CGRP is able to stimulate in situations where inotropic support is necessary (Figure 1B).

It is unclear whether the aforementioned inotropic effects of CGRP are solely a consequence CGRP directly binding to its receptor complex found on myocardial cells (Bell and McDermott, 1994), modulation of the sympathetic nervous system, or an as yet unknown mechanism. A combination of these pathways is likely, with the choice dependent on the pathophysiological setting. It is important to acknowledge that selective and non-selective β-blockers, which target β-adrenoreceptors, constitute well-established therapeutics for cardiovascular diseases such as hypertension, coronary artery disease and severe tachycardia. They decrease contractility, thus reducing cardiac output and oxygen demand (Chatterjee et al., 2013; McDonagh et al., 2021), and are therefore part of the evidence-based long-term management of heart failure with reduced ejection fraction (HFrEF). β-blockers are, however, concurrently used with ACE inhibitors, and in many cases, an aldosterone inhibitor or diuretic, to off-set other symptoms of heart failure. Despite being generally well tolerated and recommended as pharmacological therapeutics for heart failure patients who require long-term management of their medical symptoms, there remains an unmet therapeutic need for better management of HF pathophysiology.

Contrary to the above, increasing inotropy can also be of benefit in heart failure patients (Page et al., 2016). However, this is specifically for those who require inotropic- or short-term hemodynamic support due to suffering with decompensated HFrEF, presenting with low cardiac output and hypotension or evidence of end-organ hypoperfusion (McDonagh et al., 2021). Sympathetic cardiac stimulants such as dopamine and dobutamine are therefore still recommended for use in such cases (Bistola et al., 2019) and vasodilator therapeutics are also first-line agents for acute HF with elevated blood pressure (McDonagh et al., 2021). Human studies suggest that CGRP administration is protective in patients who require short-term inotropic support, whilst long-term pre-clinical studies indicate CGRP can improve cardiovascular function parameters in pressure-overload-induced heart failure.

However, whether a sub-pressor dose of CGRP can protect against the development and progression of hypertension and heart failure needs to be investigated further. If CGRP can indeed protect against cardiovascular disease without lowering blood pressure, this answers one query but raises further questions regarding its mechanism of action. Vasodilators are known to primarily reduce total peripheral resistance *via* vasodilation in blood vessels, thus lowering blood pressure. This in turn acutely enhances sympathetic stimulation due to the baroreceptor reflex, hence increasing heart rate and cardiac contractility (positive-chronotropy and -inotropy) in the short-term. The dilation of venous and arterial vessels also leads to a reduction in venous return to the heart (pre-load), which reduces congestion and after-load, therefore increasing stroke volume, cardiac output and subsequent relief of symptoms. It is for these reasons that vasodilators such as nitrates or nitroprusside are recommended for management of acute heart failure (AHF) in patients with elevated blood pressure (McDonagh et al., 2021). If a dilator such as CGRP does not affect blood pressure but is able to improve cardiac function, this adds complexity to our understanding of how vasodilators can modulate cardiac function. The recent data from Aubdool et al. (2017) and Skaria et al. (2019) suggests that CGRP can regulate cardiac function independently of blood pressure, thus *via* vascular-dependent and -independent pathways.

Collectively, these studies propose vascular-independent mechanisms for CGRP in cardiac tissues and may be the primary mechanism by which CGRP elicits protection in the absence of vascular tone changes. There is evidence supporting that CGRP can act locally on cardiomyocytes to elicit some of its cardioprotective actions, and future studies should aim to clarify the precise mechanism(s) involved in cardiac pathophysiology.

## Discussion

A wide spectrum of *in vitro, ex vivo, in vivo* and human studies have highlighted the therapeutic potential of CGRP in various pathophysiological conditions within the cardiovascular system. Genetic tools such as transgenic mouse lines combined with pharmacological agents including CGRP peptide administration systems and delivery of selective receptor antagonists, means that researchers are well-equipped to investigate the effects of reduced, enhanced and lack of CGRP signaling in whole body pathophysiology studies. More recently, anti-CGRP therapies for treatment of migraine have proven to be successful with generally minimal adverse effects reported. Additional follow-up clinical trials will be welcomed by all to clarify whether long-term CGRP blockade leads to hypertension-related side effects. Meanwhile, long lasting agonists have emerged as a promising avenue for CGRP-therapy in cardiovascular disease, which will facilitate research into the intrinsic proliferative and angiogenic characteristics of CGRP, in addition to its anti-inflammatory and anti-apoptotic effects reported *in vivo.* Future studies should aim to investigate the blood pressure-independent cardioprotective mechanisms of CGRP treatment in PO-induced heart failure, and whether treatment with the long-lasting agonist could improve outcome after ischemic heart failure. Collectively, these findings further demonstrate the importance of continuing CGRP research to fully elucidate the physiological influence of CGRP in the cardiovascular system, as well as in migraine pathophysiology.

## Author Contributions

FA wrote the first draft. Both FA and SDB finalised the manuscript. Both authors contributed to the article and approved the submitted version.

## Conflict of Interest

The authors declare that the research was conducted in the absence of any commercial or financial relationships that could be construed as a potential conflict of interest.

## Publisher’s Note

All claims expressed in this article are solely those of the authors and do not necessarily represent those of their affiliated organizations, or those of the publisher, the editors and the reviewers. Any product that may be evaluated in this article, or claim that may be made by its manufacturer, is not guaranteed or endorsed by the publisher.
